# Supplementary material for: Persistent inequality in economically optimal climate policies
Source: Nat Commun. 2021 Jun 8;12:3421. doi: 10.1038/s41467-021-23613-y (PMC8187370; doi:10.1038/s41467-021-23613-y)
Supplement: Supplementary file 1 — Supplementary Information [file 41467_2021_23613_MOESM1_ESM.pdf]

Persistent inequality in economically optimal climate policies

Supplementary Information

Paolo Gazzotti Johannes Emmerling Giacomo Marangoni Andrea Castelletti
Kaj-Ivar van der Wijst Andries Hof Massimo Tavoni

A Model regions

Regions List		
Region	Description	Countries ISO3 numeric Code
Arg	Argentina	ARG
Aus	Australia	AUS
Aut	Austria	AUT
Bel	Belgium	BEL
Bgr	Bulgaria	BGR
Blt	Baltic states	EST, LTU, LVA
Bra	Brazil	BRA
Can	Canada	CAN
Che	Switzerland	CHE
Chl	Chile	CHL
Chn	China	CHN
Cze	Czech Republic	CZE
Deu	Germany	DEU
Dnk	Denmark	DNK
Egy	Egypt	EGY
Esp	Spain	ESP
Fin	Finland	FIN
Fra	France	FRA
FSU	Former Soviet Union	ARM, AZE, BLR, GEO, KAZ, KGZ, MDA, TJK, TKM, UZB
GBR	UK	GBR
Gulf	Gulf Countries	ARE, BHR, IRN, IRQ, KWT, OMN, QAT, SAU, YEM
Grc	Greece	GRC
Hrv	Croatia	HRV
Hun	Hungary	HUN
Idn	Indonesia	IDN
Ind	India	IND
Irl	Ireland	IRL
ita	Italy	ITA
jpn	Japan	JPN

Kor	Korea	KOR
MEast	Middle East	ISR, JOR, SYR, LBN, PSE
Mex	Mexico	MEX
Mys	Malaysia	MYS
Nld	Netherlands	NLD
NAfr	North Africa	ESH, TUN, MAR
NWAfr	North-West Africa	LBY, DZA
Nor	Norway	NOR
Ocean	Pacific Island	CXR, COK, HMD, NFK, NIU, NRU, PCN, TKL, TUV, UMI, WLF, FJI, PNG, FSM, GUM, ASM, TLS, PYF, KIR, MNP, MHL, NCL, PLW, WSM, SLB, TON, VUT, NZL
Pol	Poland	POL
Prt	Portugal	PRT
RCAm	Rest Central America	BES, CUW, SXM, ABW, BHS, BLZ, BRB, CRI, CUB, DMA, DOM, GRD, GTM, HND, HTI, JAM, LCA, NIC, PAN, SLV, TTO, VCT, BMU, SGS, TCA, VGB, VIR, AIA, ATG, BLM, CYM, GLP, KNA, MAF, MSR, MTQ, PRI
REur	Rest Europe	CYP, LUX, MLT, LIE, GRL, ISL, FRO, ALA, AND, GGY, GIB, IMN, JEY, MCO, SJM, SMR, VAT, SPM, BIH, ALB, MKD, MNE, SRB, KSV
Rou	Romania	ROU
RSAm	Rest South America	BOL, COL, ECU, FLK, GUF, GUY, PER, PRY, SUR, URY, VEN
RSAs	Rest South Asia	AFG, BGD, BTN, LKA, MDV, NPL, PAK
RSEAs	Rest South-East Asia	BRN, CCK, KHM, LAO, MMR, PHL, SGP, PRK, HKG, MAC, TWN, MNG
Rus	Russia	RUS
SSAfr	Sub-Saharan Africa	AGO, BEN, BWA, BFA, BDI, CMR, CPV, CAF, TCD, COM, COG, COD, CIV, GNQ, ERI, ETH, GAB, GMB, GHA, GIN, GNB, KEN, LSO, LBR, MDG, MWI, MLI, MRT, MUS, MYT, MOZ, NAM, NER, NGA, REU, RWA, STP, SEN, SYC, SHN, SLE, SOM, SSD, SDN, SWZ, TZA, TGO, UGA, ZMB, ZWE, DJI, IOT, BVT, ATF
Slo	Slovenia	SVN
Svk	Slovakia	SVK
Swe	Sweden	SWE
Tha	Thailand	THA
Tur	Turkey	TUR
Ukr	Ukraine	UKR
USA	USA	USA
Vnm	Vietnam	VNM
Zaf	South Africa	ZAF

B Additional figures

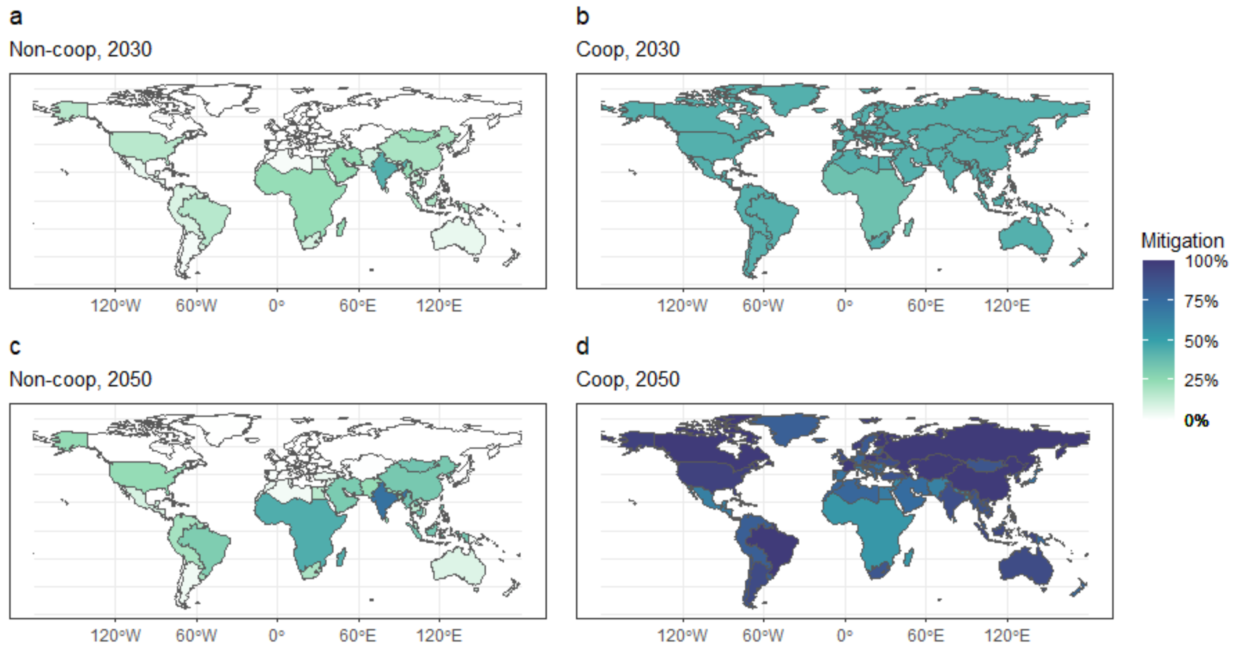

Supplementary Figure 1: **Mitigation effort map in 2030 and 2050.** Values refer to percentage of baseline emissions reduced, under the pathway SSP2 and BHM-SR impact specification. Cooperation uses an intermediate inequality aversion level ($\gamma = 0.5$).

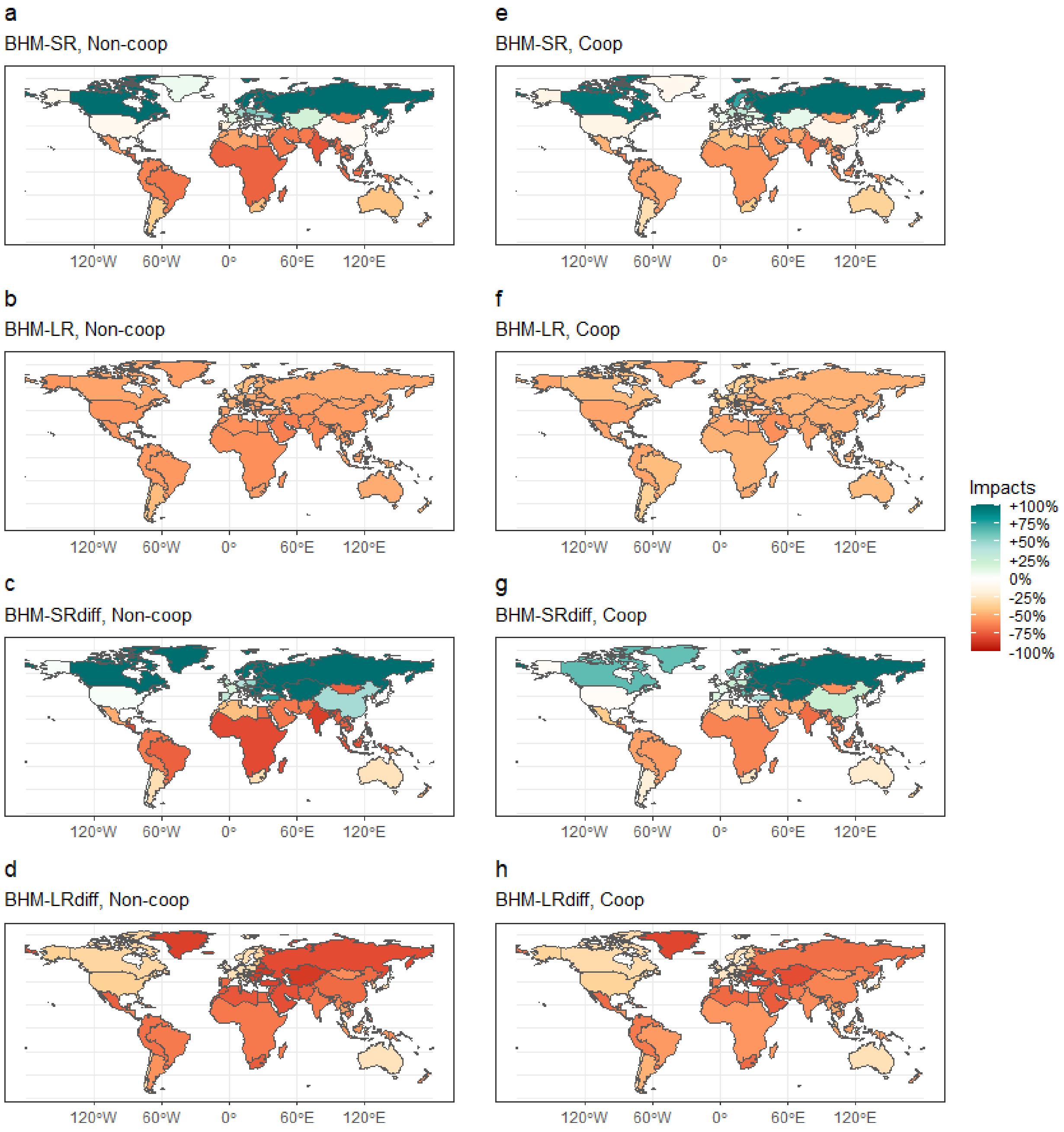

Supplementary Figure 2: **Climate impacts distribution in 2100. a-d,** Non-cooperative scenario. **e-h,** Cooperative scenario with intermediate inequality aversion ($\gamma = 0.5$), socioeconomic pathway SSP2. All four BHM impact-function specifications are shown.

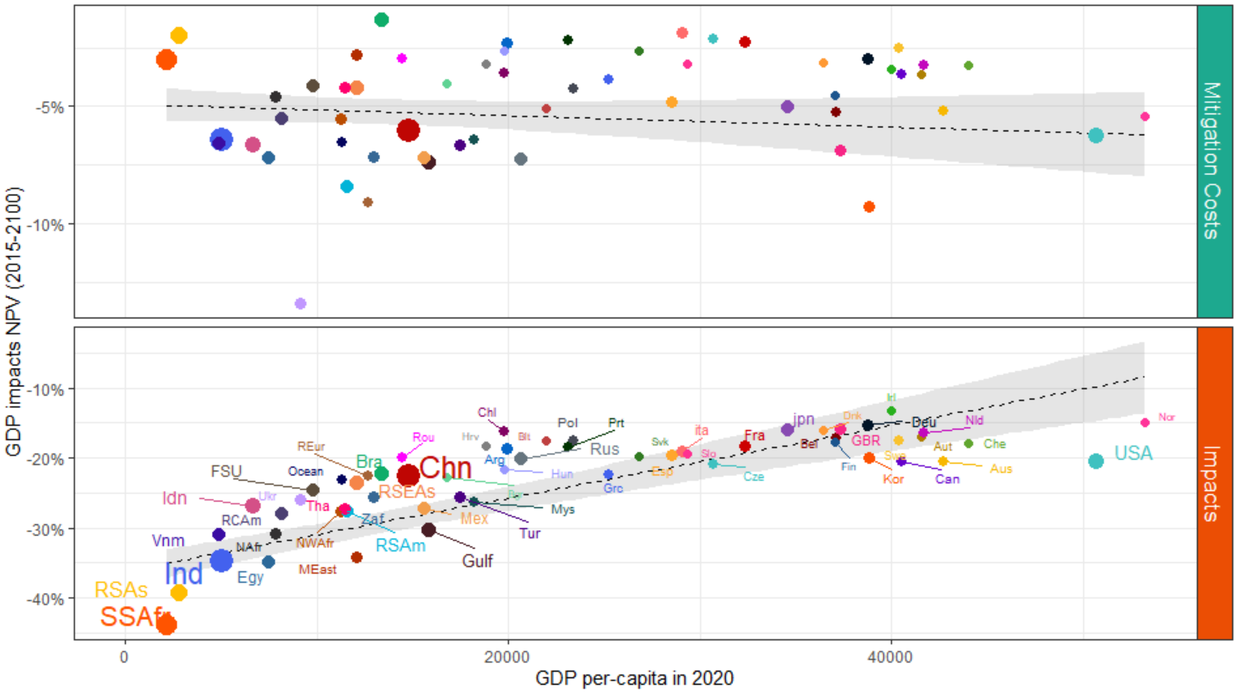

Supplementary Figure 3: GDP net present value impacts and costs against 2020 GDP per-capita under BHM-LR impacts. NPV accounts for years 2015-2100, with world-averaged Ramsey yearly discount rate. Reported scenario is cooperative, SSP2, under BHM-LR impact function and intermediate levels for inequality aversion ($\gamma = 0.5$) and utility discount rate ($\rho = 1.5\%$).

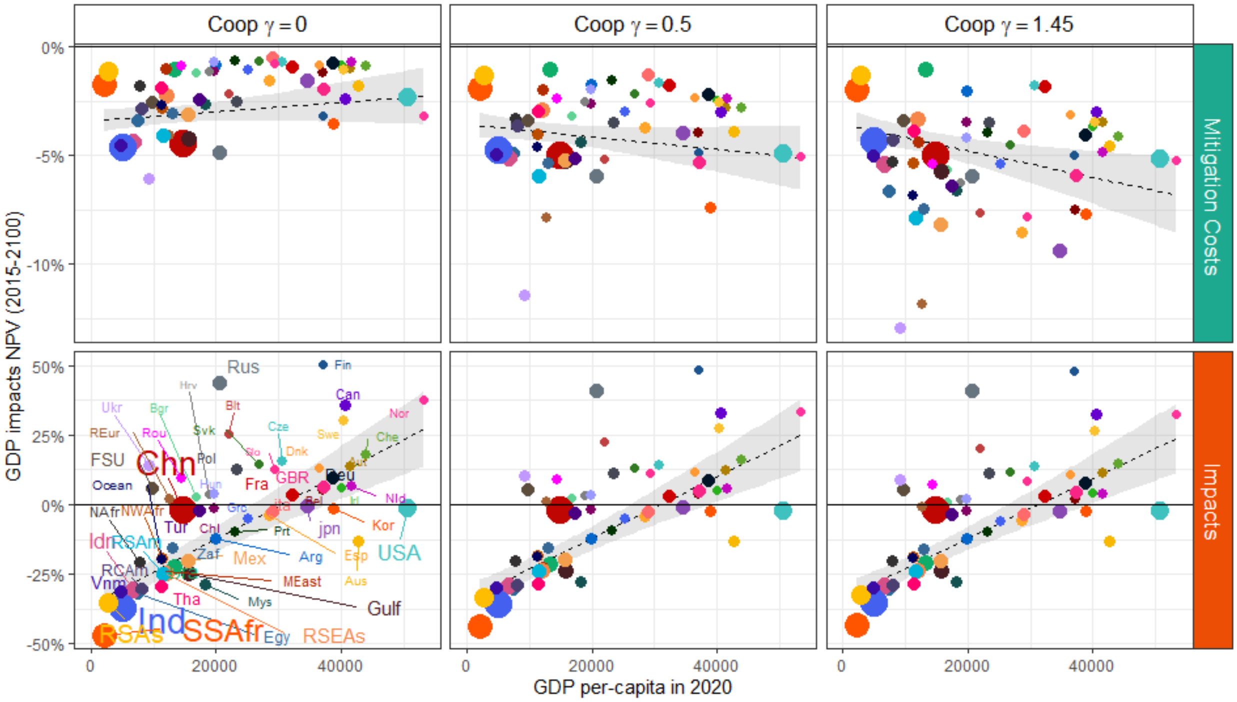

Supplementary Figure 4: GDP net present value impacts and costs against 2020 GDP per-capita under alternative inequality aversion values. NPV accounts for years 2015-2100, with world-averaged Ramsey yearly discount rate. Reported scenario is cooperative, SSP2, under BHM-SR impact function, intermediate level for utility discount rate ($\rho = 1.5\%$) and different representative values of inequality aversion γ.

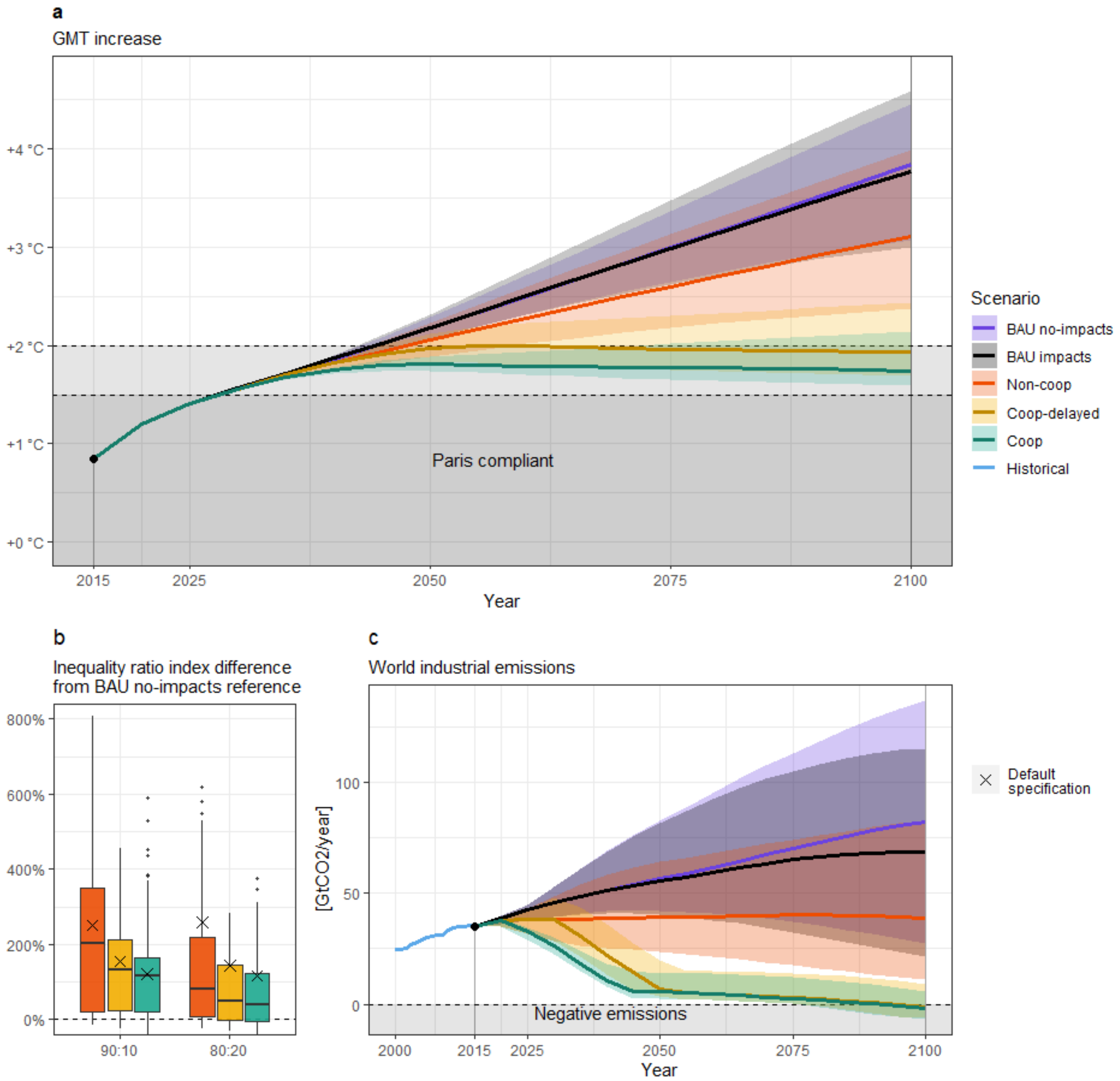

Supplementary Figure 5: **Optimal outcomes with delayed cooperation. a,** Global Mean Temperature (GMT) increase over time. **b,** Inequality index relative difference from BAU-no-impacts for Non-coop, Coop-delayed, and Coop scenarios. **c,** World-aggregated CO_2 emissions trends with delayed cooperation. Uncertainty ranges include SSP projection and impact specification. Both Coop and Coop-delayed scenarios are with intermediate inequality aversion ($\gamma = 0.5$). Delayed cooperation starts in 2030.

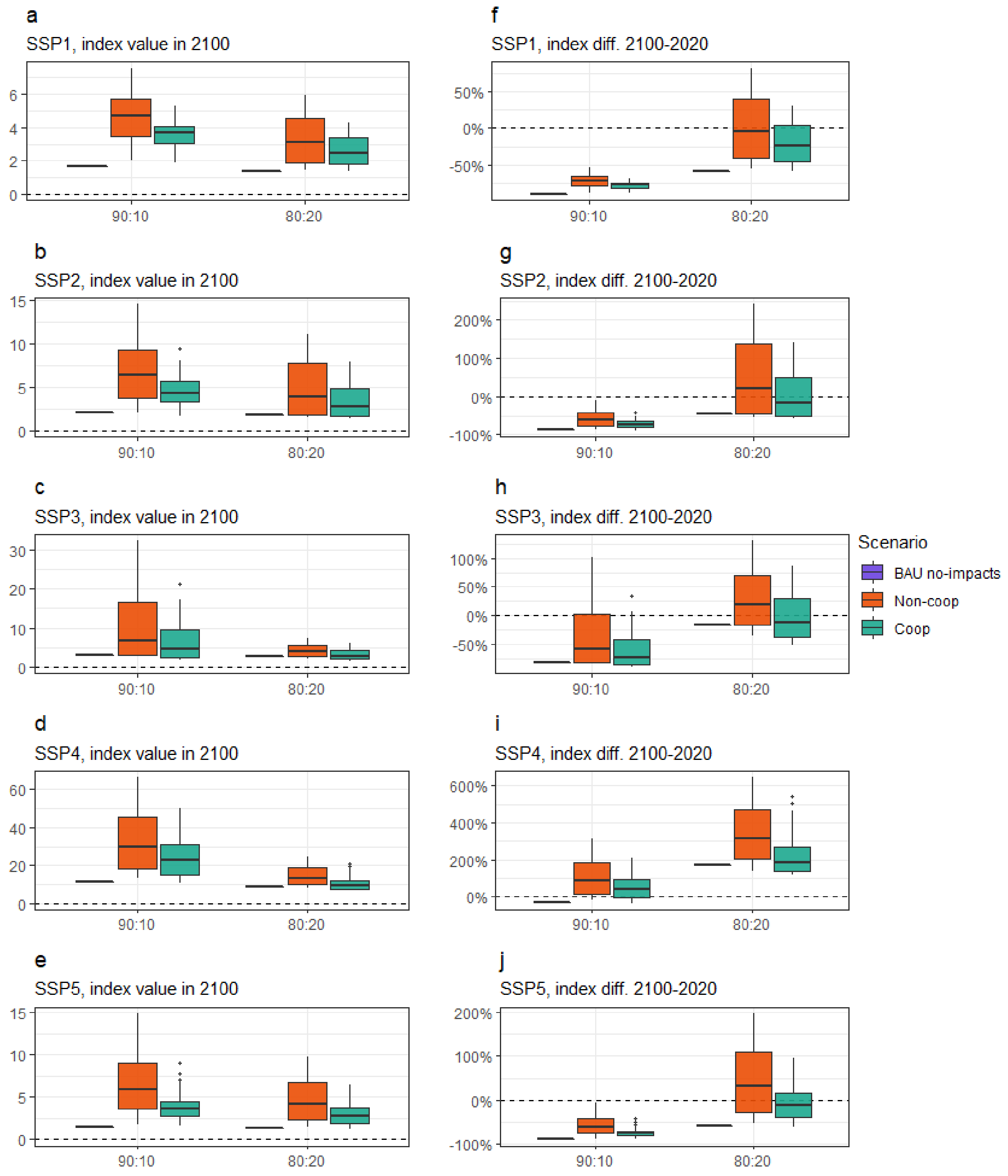

Supplementary Figure 6: **Inequality index distribution in 2100 for each SSP reference. a-e,** Indexes value distribution. **f-j,** Indexes percentage difference from 2020 values: 15.93 (90:10 ratio) and 3.24 (80:20 ratio).

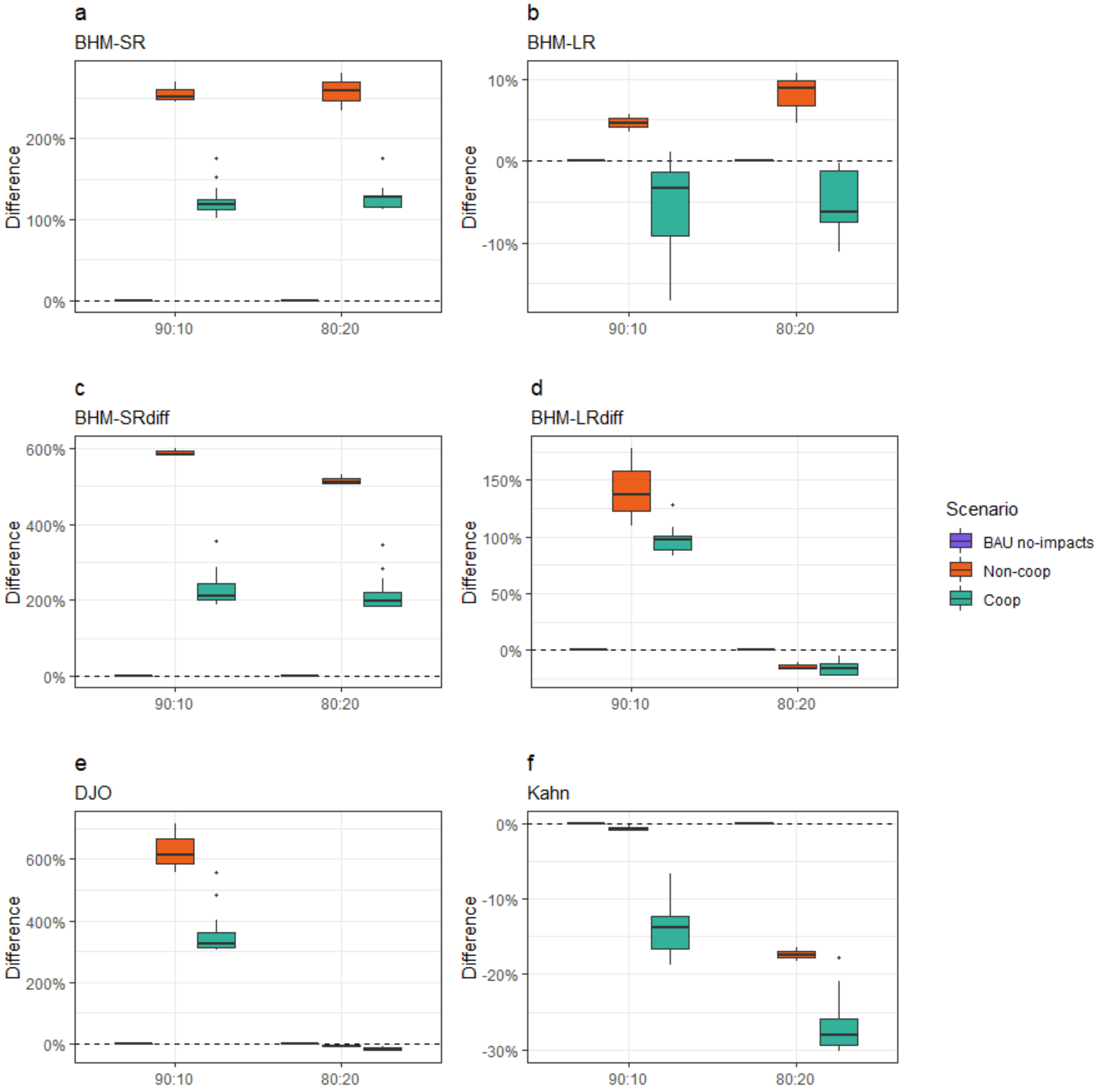

Supplementary Figure 7: **Inequality index distribution in 2100 for each impact specification. a-d,** BHM main impact specifications. **e,** Robustness analysis with DJO impact specification. **f,** Robustness analysis with Kahn impact specification. All values refer to percentage difference from BAU-no-impact references.

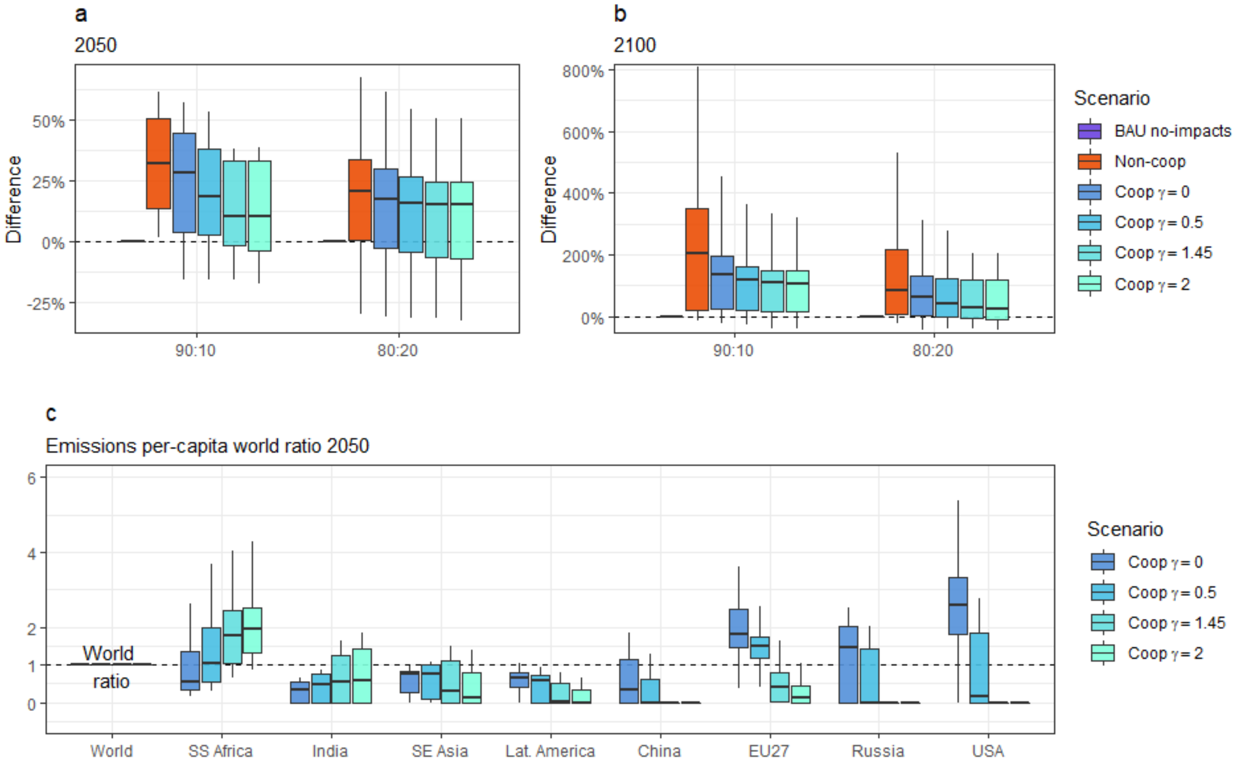

Supplementary Figure 8: **Inequality aversion effect on inequality and emissions. a,** Inequality index relative difference from BAU-no-impacts for Non-coop and Coop under increasing inequality aversion in 2050. **b,** Inequality index relative difference from BAU-no-impacts for Non-coop and Coop under increasing inequality aversion in 2100. **c,** Ratio between main regions and world emissions per-capita distribution under Coop at increasing inequality aversion. They show the aggregated level from finer geographical-resolution results.

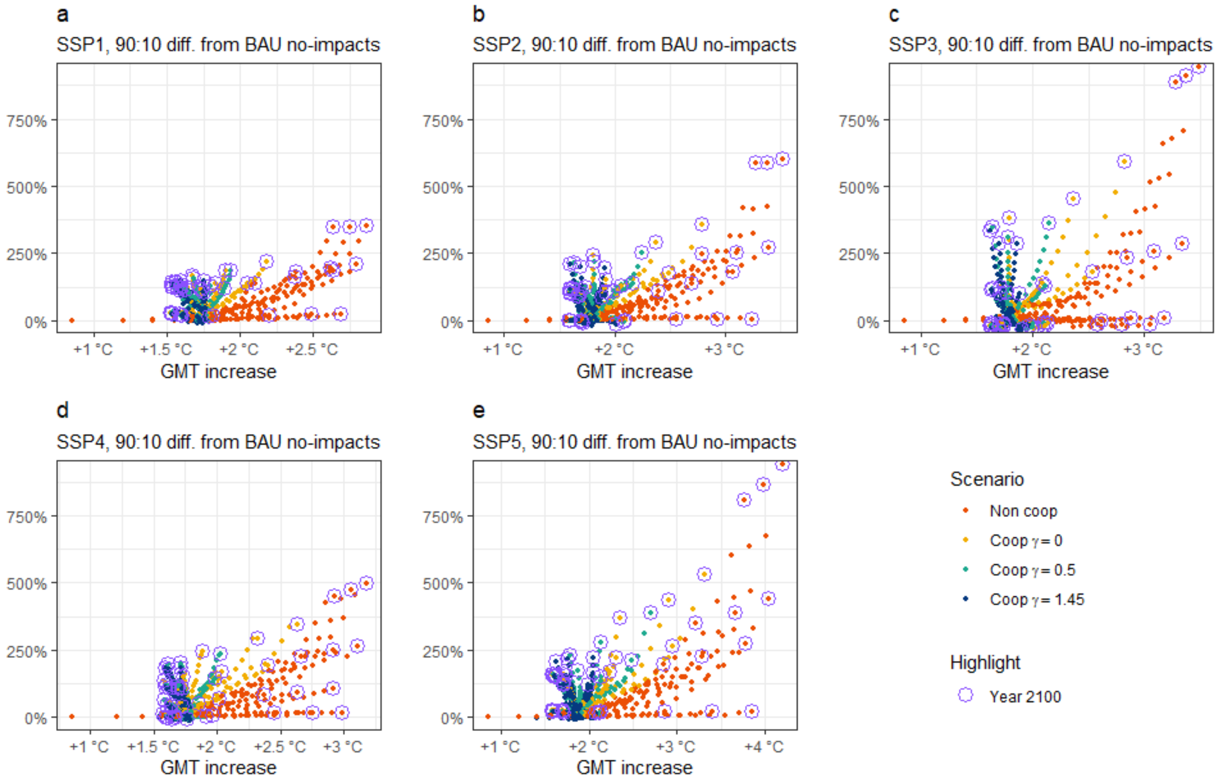

Supplementary Figure 9: **Ratio 90:10 inequality index over GMT increase, for each SSP scenario.** The y-axis shows percentage difference from BAU-no-impacts levels. Time dimension (2015-2100) is hidden in data, but 2100 levels are highlighted. Colors show also different levels of inequality aversion.

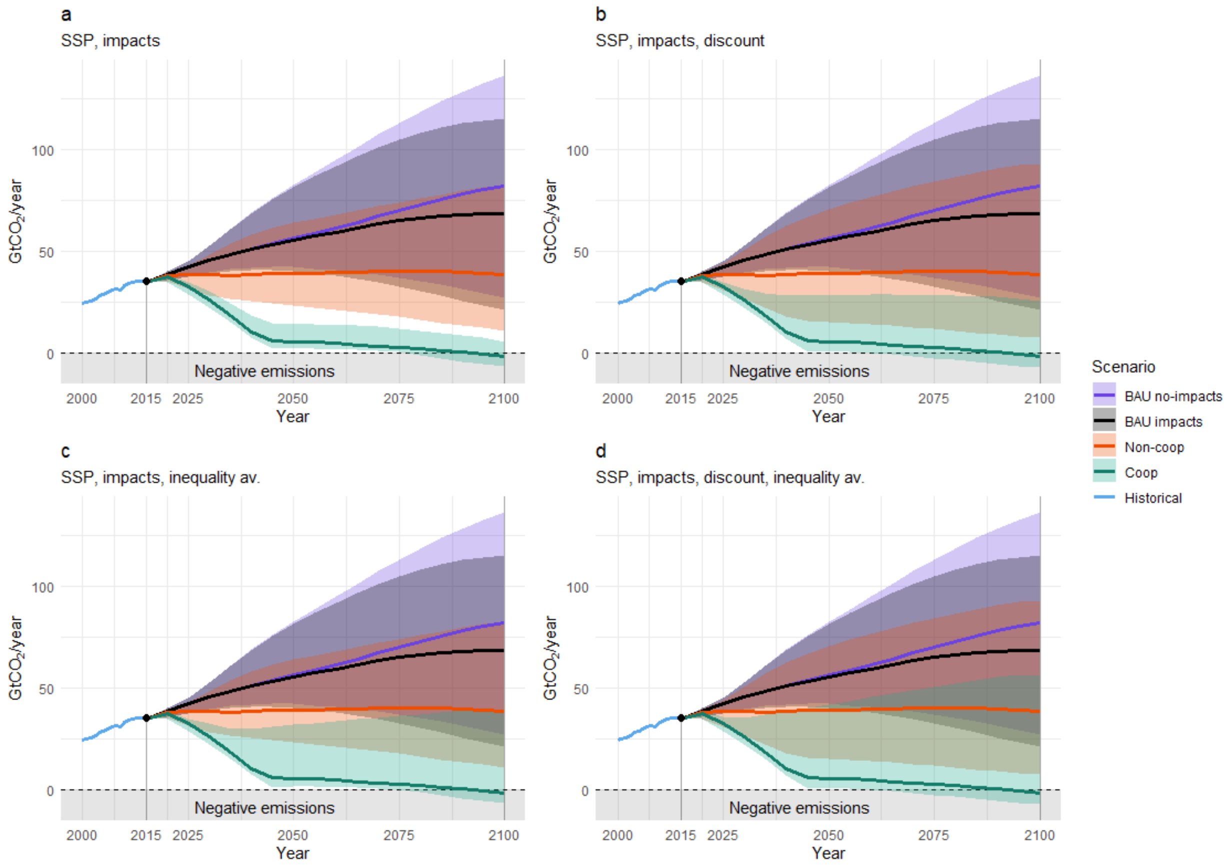

Supplementary Figure 10: **Optimal world-aggregated model emissions with different uncertainty ranges. a,** Uncertainty ranges include SSP baselines and impact definitions (as in Figure 1a). **b,** Uncertainty ranges include SSP baselines, impact definitions and utility discount rate. **c,** Uncertainty ranges include SSP baselines, impact definitions and inequality aversion. **d,** Uncertainty ranges include all factors: SSP baselines, impact definitions, utility discount rate and inequality aversion.

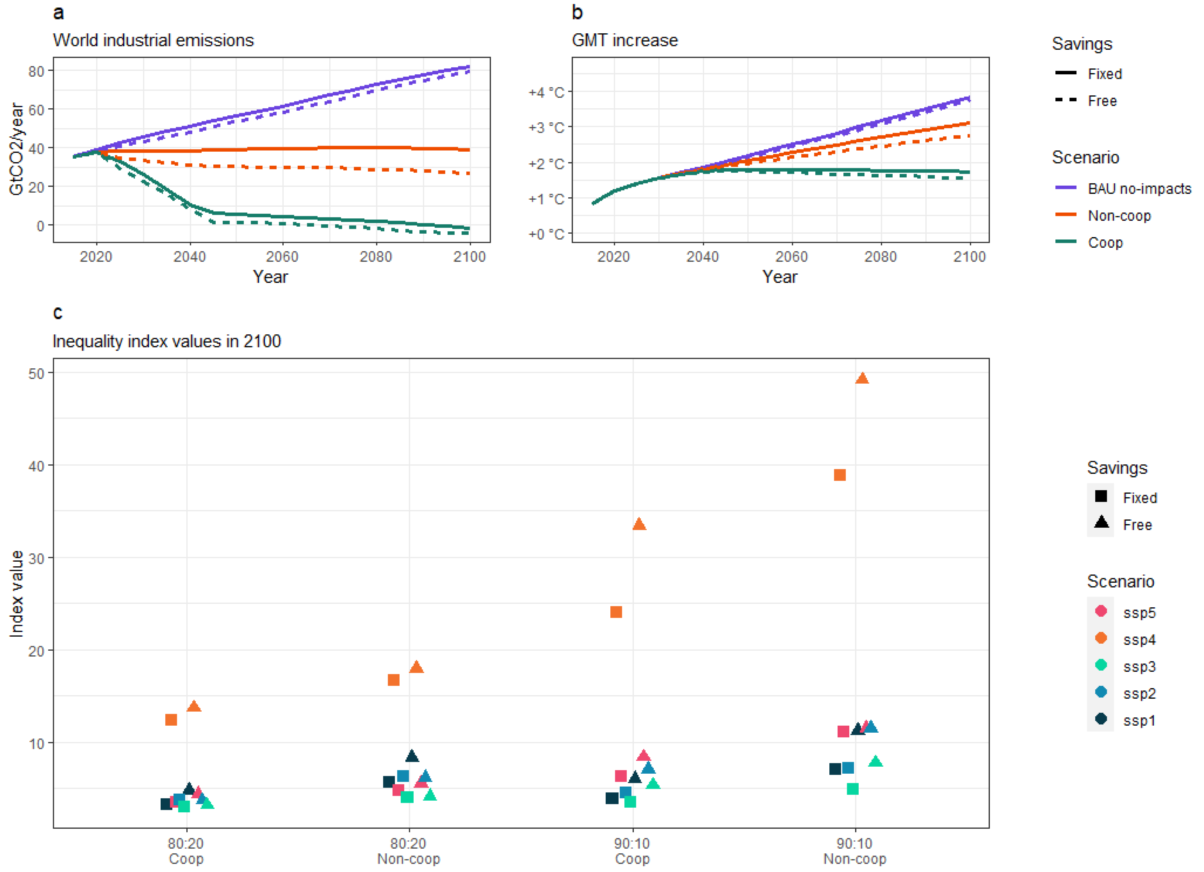

Supplementary Figure 11: **Comparison between fixed and endogenous savings rate solutions. a-b,** World emissions and GMT increase for SSP2 baseline and main cooperation options. **c,** Inequality indexes comparison across all SSP scenarios both for cooperative and non-cooperative solutions.
